# Targeting the RdRp of Emerging RNA Viruses: The Structure-Based Drug Design Challenge

**DOI:** 10.3390/molecules25235695

**Published:** 2020-12-03

**Authors:** Francesca Picarazzi, Ilaria Vicenti, Francesco Saladini, Maurizio Zazzi, Mattia Mori

**Affiliations:** 1 Department of Biotechnology, Chemistry and Pharmacy, Department of Excellence 2018–2022, University of Siena, Via Aldo Moro 2, 53100 Siena, Italy; francesca.picarazzi@student.unisi.it; 2 Department of Medical Biotechnologies, University of Siena, 53100 Siena, Italy; vicenti@unisi.it (I.V.); saladini6@unisi.it (F.S.); maurizio.zazzi@unisi.it (M.Z.)

**Keywords:** RdRp, Mg^2+^ ions catalysis, emerging RNA viruses, small molecule inhibitors, structure-based drug design

## Abstract

The RNA-dependent RNA polymerase (RdRp) is an essential enzyme for the viral replication process, catalyzing the viral RNA synthesis using a metal ion-dependent mechanism. In recent years, RdRp has emerged as an optimal target for the development of antiviral drugs, as demonstrated by recent approvals of sofosbuvir and remdesivir against Hepatitis C virus (HCV) and severe acute respiratory syndrome coronavirus 2 (SARS-CoV-2), respectively. In this work, we overview the main sequence and structural features of the RdRp of emerging RNA viruses such as Coronaviruses, Flaviviruses, and HCV, as well as inhibition strategies implemented so far. While analyzing the structural information available on the RdRp of emerging RNA viruses, we provide examples of success stories such as for HCV and SARS-CoV-2. In contrast, Flaviviruses’ story has raised attention about how the lack of structural details on catalytically-competent or ligand-bound RdRp strongly hampers the application of structure-based drug design, either in repurposing and conventional approaches.

## 1. Introduction

**Figure 1:**
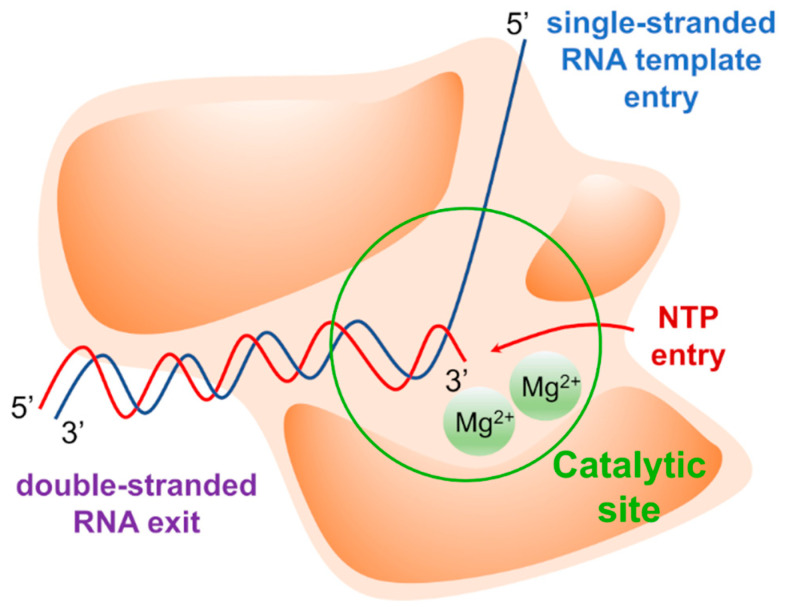
Graphical representation of the RdRp. The RNA template is represented by the blue line. The nascent RNA is represented by the red line. Mg^2+^ ions are represented as green spheres.

The replication of the viral genome is a complex mechanism in which several viral and host factors are involved. Once delivered into the host cell following virus entry and uncoating, the viral genome directs its own replication as well as translation of viral proteins. Most viruses coding for an RNA-dependent RNA polymerase (RdRp) complete these processes in the cytoplasm, with some notable exceptions (e.g., influenza virus). RdRp is a multi-domain viral enzyme, encoded by all RNA virus families except for *Retroviridae*, which is classified as a transferase (EC 2.7.7.48) [1]. RdRp catalyzes the synthesis of a nascent RNA strand by adding ribonucleotide units to the 3’-hydroxyl terminus, building the RNA molecule in the 5’-3’ direction. To carry out its polymerase activity, RdRp requires an RNA template, ribonucleotide 5’ triphosphates (ATP, GTP, UTP, and CTP) as precursors of the nucleotide units of nascent RNA, and two magnesium ions (Mg^2+^) within the active site that catalyze the phosphodiester-bond formation. The polymerase may also bind zinc ions (Zn^2+^) with tetrahedral coordination in a site that is located outside the catalytic site, where these ions play a structural role [2,3,4]. The structural arrangement of the RdRp forms two channels that meet at the active site. The main channel accommodates the RNA template, while the secondary channel allows the inclusion of incoming nucleotides triphosphate (NTPs) (Figure 1).

**Figure 2:**
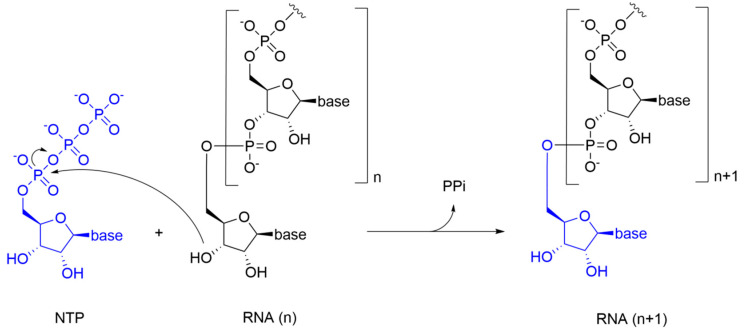
RNA synthesis reaction catalyzed by the RdRp.

NTPs of the newly synthesized RNA are inserted according to Watson-Crick base pairing rules: U/A is inserted into the nascent RNA strand to pair with A/U from the RNA template, while G/C is inserted to pair with C/G. In the catalytic reaction, the 3’-hydroxyl group of the nascent RNA acts as a nucleophile, attacking the α-phosphate of the incoming NTP and releasing a pyrophosphate molecule (PPi) (Figure 2). 

This process is promoted by Mg^2+^ ions that are coordinated with an octahedral geometry by the phosphate groups of the incoming NTP, and by the three aspartate residues that are highly conserved among different viral RdRps. Specifically, a Mg^2+^ ion promotes the nucleophilic attack of the 3’-hydroxyl group from the nascent RNA to the α-phosphate of the incoming NTP, while the other Mg^2+^ ion facilitates the detachment of the PPi molecule. This reaction mechanism is shared by all RdRps, and it is commonly referred to as the “two metal ions catalysis” [5]. 

The RdRp can initiate RNA synthesis from either the end of the RNA template, or by recognizing an internal promoter sequence [6]. Mutagenic studies have shown that alterations in the secondary structure of RNA can affect its interaction with the RdRp and interfere with the catalytic process [7].

Since RNA viruses have a relatively high mutation rate in the order of 10^−6^–10^−4^ substitutions per nucleotide site per cell infection [8], they have evolved by developing various proofreading mechanisms. Indeed, viral replication can be an imprecise and discontinuous process. The rapid insertion of bases during RNA elongation may be subject to misincorporation of mismatched nucleotides, leading to RdRp dysfunction. For this reason, the elongation process can be interrupted by a backtracked pause. During a backtracked pause, two events occur: (i) the RdRp moves upstream, allowing the exit of the 3’-end of the RNA transcript through the NTP channel; and (ii) cleavage of the most recently incorporated ribonucleotides, which restore catalytic functions [9]. Backtracking has been extensively studied in eukaryotes and in some bacteria, such as *Escherichia coli*, and only recently has been observed in viral RdRps. However, it remains a very rare process [10]. Differently from other RNA viruses, the fidelity of CoV RdRp is increased by a 3’ to 5’ exonuclease (NSP14) that exerts a proofreading function during RNA synthesis. NSP14 is highly conserved in the *Coronoviridae* family and is considered an essential factor for the existence and evolution of large RNA genomes such as those of CoVs, by avoiding lethal mutagenesis and maintaining replication competence [11].

Considering that RdRp plays a crucial role in the replication cycle of most RNA viruses, its high conservation among evolutionary distant RNA viruses [12], the absence of RdRp homologous in mammalian cells, the extensive knowledge on RdRp structure and functions, and the easy development and consequent availability of biochemical assays for the rapid screening of large libraries of compounds, RdRp is considered as an attractive target for the discovery of novel antiviral drugs. Nevertheless, the emergence of RdRp drug-resistant variants might limit the broad application of specific inhibitors, or might require their use in combination with drugs directed to other viral targets, if available. Successful examples of RdRp inhibitors include sofosbuvir, which is clinically approved for the therapy of Hepatitis C virus (HCV) infection, and remdesivir, which was originally designed as a therapy for Ebola virus infection and has been recently approved for the treatment of hospitalized patients with severe coronavirus disease 2019 (COVID-19).

In this review, we summarize the structural details that characterize the RdRp of emerging RNA viruses such as Severe Acute Respiratory Syndrome coronavirus (SARS-CoV), Severe Acute Respiratory Syndrome coronavirus-2 (SARS-CoV-2), Middle East Respiratory Syndrome coronavirus (MERS-CoV), Zika virus (ZIKV), West Nile virus (WNV), Dengue virus (DENV), and HCV. Moreover, we revise the genetics and pathogenicity of these viruses and discuss the main RdRp inhibition strategies developed so far. Finally, we describe the state of the art in drug design of new RdRp inhibitors through the structure-based approach, shedding light on future directions and perspectives.

## 2. RNA Virus Outbreaks

Although the number of RNA viruses that have caused outbreaks in recent years is relatively high, here we focus on those viruses that we believe have the strongest clinical relevance, social impact, and scientific interest. The pandemic outbreak of the new coronavirus is threatening the health systems on a global scale, and the scientific community is making an unprecedented multidisciplinary effort to face the urgent need for both treatment and prevention of severe acute respiratory syndrome coronavirus 2 (SARS-CoV-2) infections. On the other hand, HCV is a notable example of how coordinated and extensive research efforts can bring successful results in the development of potent and well-tolerated specific antiviral drugs. Both HCV and SARS-CoV-2 case studies might inspire the development of drugs against RNA viruses for which no effective cures are yet available despite high pathogenicity for humans. 

### 2.1. Coronaviruses’ Outbreaks

At the end of 2002, a new respiratory disease named Severe Acute Respiratory Syndrome (SARS), caused by the previously unknown coronavirus SARS-CoV, was detected in the province of Guangdong, China. This new virus rapidly affected China and other countries, including several countries in South East Asia, Canada, and Europe [13]. In 2012, in the Middle East, a new coronavirus named MERS-CoV appeared as the causative agent of the Middle East Respiratory Syndrome (MERS). MERS cases were reported predominantly in Saudi Arabia, the United Arab Emirates, Qatar, Oman, and Kuwait, while occasional cases were imported in Europe [14]. Despite these two outbreaks, the scientific community and international health agencies have continued to take a marginal interest in these viruses, as they never became pandemic and the number of cases was relatively low. Along with the disappearance of these two potentially lethal coronaviruses, research and development of specific antiviral agent and preventive vaccines were discontinued early [15]. In December 2019, the outbreak of a new coronavirus in Wuhan, China, formerly known as 2019-nCoV and later renamed as SARS-CoV-2, caused a new respiratory syndrome also known as COVID-19, classified as a pandemic by the WHO in February 2020. COVID-19 is currently representing a major health issue worldwide, and the search for effective cures and vaccines has rapidly become a top priority, not only to arrest virus related fatality, but also to resume the global economy put on hold by lockdown measures set to contain the epidemic.

### 2.2. Flaviviruses’ Outbreaks

Flaviviruses are transmitted through the bite of infected mosquitoes. Specifically, ZIKV and DENV use mosquitoes of the Aedes genus as a vector, while WNV uses those from the Culex genus. ZIKV was first identified in Uganda in 1947 [16,17,18]. Since the mid-20th century, ZIKV has spread in many African regions [19,20], Southeast Asia [21,22], and South America, especially in Brazil, where it caused a severe outbreak in 2016 [23,24]. Moreover, ZIKV infection is a major threat during pregnancy, as the virus can be transmitted vertically and cause microcephaly [25,26]. DENV followed a similar spread-scheme as ZIKV [27]. Its first identification dates back to 1943 in Japan [28,29]. From the second half of the 20th century, DENV spread particularly in Latin America and Southeast Asia, and even reached Europe [30,31]. WNV is endemic in different areas such as Africa [32], Southeast Europe [33,34], United States [35,36], Australia [37] and Middle East. Although these viruses are spreading all over the world with variable clinical consequences, there is no specific drug to treat at least the most severe infections. Indeed, therapy is merely supportive, and clinical benefits strongly depend on early diagnosis and timely intervention. As a further complication, all the diseases caused by these Flaviviruses share initial symptoms like rash, vomiting, headache, and fever, thus making it difficult to distinguish among them [38]. 

### 2.3. HCV Outbreak

First identified in 1989, but long known as the elusive agent of non-A non-B hepatitis, HCV is a worldwide-spread virus [39] that includes eight genotypes and many subtypes which are distinguished by geographic distribution, transmission route, and rate of disease progression [40]. HCV specifically infects liver cells, and it is transmitted through infected blood direct contacts, often due to the use of intravenous drugs, unsterilized medical devices or blood transfusions [41]. There is no vaccine for the prevention of HCV infection. However, testing donated blood and implementing safe procedures in the healthcare setting has greatly reduced HCV incidence [42]. To date, licensed drugs that can eradicate HCV infection are available, and the World Health Program to eliminate HCV by 2030 is progressing with variable success, with diagnosing and treating all HCV carriers remaining the highest challenge. The advent of these drugs, including NS3 protease inhibitors, NS5A inhibitors, and one nucleoside analog RdRp inhibitor (sofosbuvir) has dramatically changed the fate of chronic HCV infection, avoiding long-term complications such as liver cirrhosis and hepatocellular carcinoma [43,44]. Many resources have been invested in the study of this virus and sofosbuvir is a key example in the development and testing of highly successful RdRp inhibitors.

## 3. Sequence of Target RNA Viruses

Since RNA viruses are highly polymorphic, continuous surveillance is warranted to detect novel variants and spread to new geographic areas or patient populations. Thus, updating knowledge of RNA viruses such as SARS-CoV, SARS-CoV-2, MERS-CoV, ZIKV, WNV, DENV, and HCV remains a challenge. In addition, veterinary virology is an integral part of this activity to support the capability to characterize zoonotic viruses crossing the species barrier and entering the human population. Once a novel human pathogen is suspected or identified, obtaining partial or full genome information is a major step towards the management of the disease and epidemic. A notable example of this kind is SARS-CoV-2, which was detected via a metagenomic approach, sequenced and classified as a new member of the *Coronaviridae* family based on sequence homology [45]. Following further sequence analysis, this “novel coronavirus 2019” (2019-nCoV), was finally recognized as the second variant of SARS-CoV and thus renamed SARS-CoV-2.

Undoubtedly, detection of novel virus variants and species has been facilitated by the dramatic progress in whole-genome sequencing [46,47,48,49,50,51], thanks to the advent of a number of next-generation sequencing methods allowing full-length sequencing at a fraction of the time and cost required for traditional Sanger sequencing [52]. 

**Figure 3:**
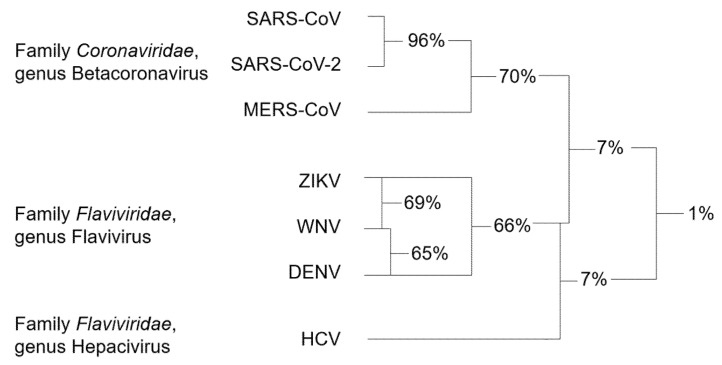
Schematic representation of the percentages of identity of the RdRp shared by RNA viruses of the same cluster and between different clusters.

Genetic analysis of the RNA viruses considered in this review led to a systematic classification of the different species with respect to the RdRp sequence. Accordingly, RdRp sequences can be divided into three clusters, which are notably related to the family and genus they belong to (Figure 3).

**Figure 4:**
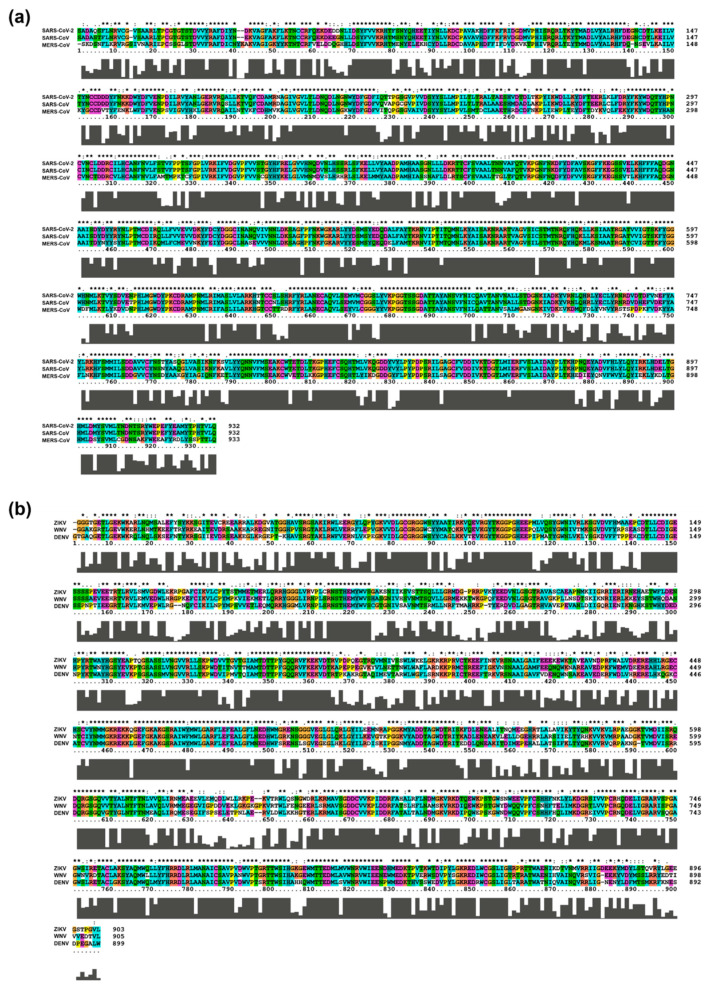
Alignment of RdRp sequences from members of the (**a**) Betacoronavirus and (**b**) Flavivirus genus. The sequences alignment was carried out using the ClustalX version 2.1 [54] with the pairwise alignment algorithm, keeping the default color scheme for the amino acids, i.e., blue = hydrophobic; red = positive charge; magenta = negative charge; green = polar; pink = cysteine; orange = glycine; yellow = proline; cyan = aromatic; white = unconserved or gaps. The black bar below the alignment corresponds to the level of conservation: the higher the bar, the higher the conservation at every position. * indicates the amino acids that are conserved in the aligned sequences.

Among Coronaviruses, SARS-CoV-2 RdRp shares the highest amino acid identity (96%) with SARS-CoV RdRp, while homology with MERS-CoV RdRp is only 70%. The average sequence identity is lower among flaviviruses, with ZIKV, WNV, and DENV RdRps sharing 66–69% of amino acids. HCV, belonging to a different genus of the *Flaviviridae* family, has very limited homology with the members of the *Flavivirus* genus [53]. For this reason, HCV RdRp was not included in the sequence alignment of Figure 4.

Although whole-genome sequencing is the key strategy for tracking outbreaks and virus evolution, genome analysis is only the first step to design and test antiviral candidates. Indeed, structural details are essential to assess the druggability of a potential target protein, and they might be provided by structural biology efforts or, to a more immediate but less precise extent, by homology modeling. While some structural information is available for HCV and coronaviruses (see next chapters), in the case of flaviviruses, the lack of structural details on catalytically-competent RdRp strongly hampers the design of new specific inhibitors, often making it necessary to build homology models with all the complications derived from a low percentage of identity [55].

## 4. Structural Features of RdRp from Emerging RNA Viruses

**Figure 5:**
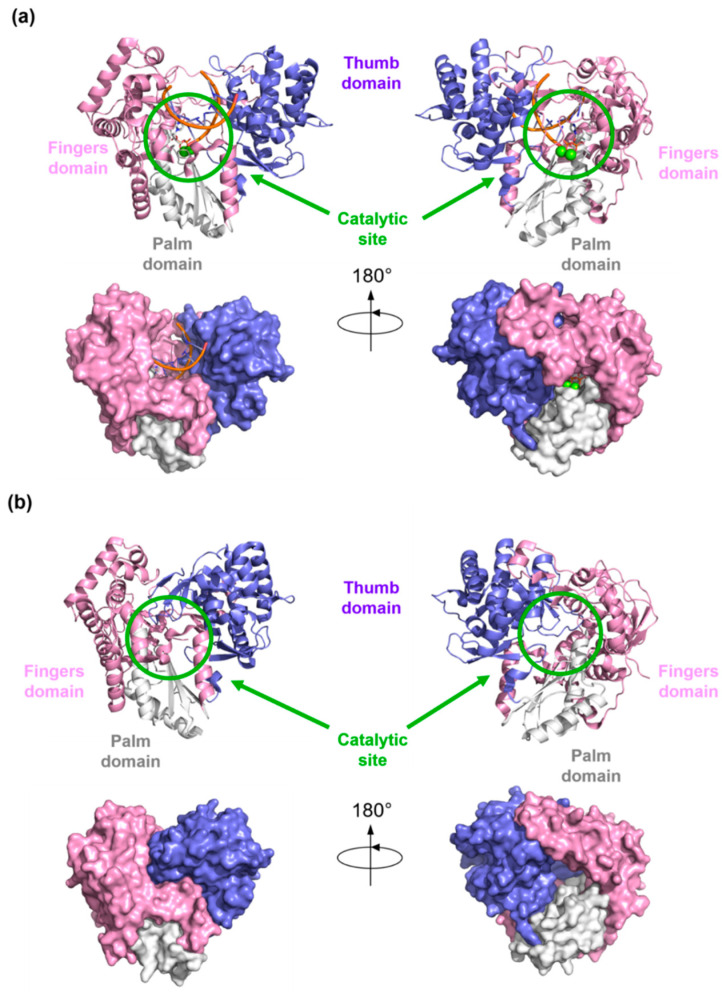
Graphical representations of palm (white), thumb (blue) and fingers (pink) subdomains in (**a**) RNA-bound RdRp (PDB-ID: 4WTG) and (**b**) *apo* RdRp (PDB-ID: 2XI2). The protein structures are represented as cartoons and as surfaces in different orientations. The catalytic site is highlighted by the green circle, and the metal ions are represented as green spheres.

Structural details of viral RdRp collected by X-Ray crystallography, NMR, or Cryo-EM techniques, give access to a great understanding of the structural architecture of this protein. Overall, the shape of the viral RdRp is comparable to a closed right hand with palm, thumb, and fingers subdomain (Figure 5).

The fingers subdomain has been further divided into index, middle, ring, and pinky fingers. [56,57]. The palm domain is the most conserved among the RdRp of different viral species and contains the catalytic site [53,58,59,60,61]. It is composed of five catalytic motifs (A–E), of which A and C have conserved aspartic acid residues that participate in the catalytic reaction by coordinating the Mg^2+^ ions [62]. Fingers and thumb subdomains are located at the N- and C-terminus of the RdRp, respectively, and form two tunnels that meet at the active site: one tunnel is for the access of the NTPs into the catalytic site, while the other crosses the whole polymerase and allows the entry of RNA template and the exit of double-stranded RNA (Figure 1). Moreover, each species has specific N-terminal and C-terminal tail domains or cofactors, such as the nonstructural proteins (NSPs) 7 and 8 in Coronaviruses that increase the polymerase activity, and the domain with methyltransferase (MTase) activity in Flaviviruses and HCV.

### 4.1. Structure of Coronaviruses RdRp

**Figure 6:**
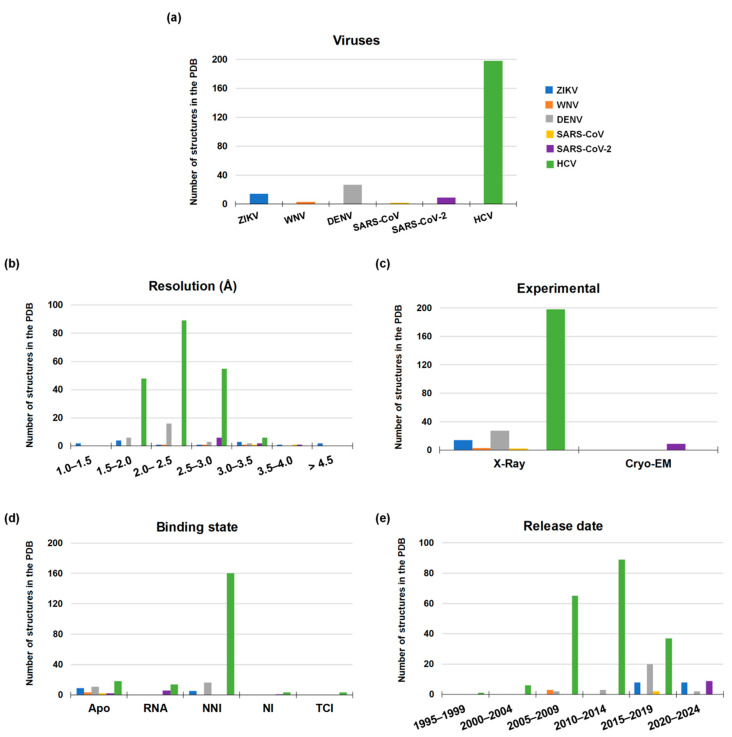
Statistics of three-dimensional structures available for SARS-CoV, SARS-CoV-2, ZIKV, WNV, DENV and HCV RdRp in the Protein Data Bank divided by (**a**) viruses, (**b**) resolution, (**c**) experimental methods, (**d**) binding state and (**e**) release date. Abbreviation used in (**d**): Targeted covalent inhibitor (TCI), nucleos(t)ide analogue inhibitor (NI), non-nucleos(t)ide inhibitor (NNI).

Coronaviruses are positive-strand RNA viruses. Their genome has two large open reading frames (ORF1a and ORF1ab) encoding for two polyproteins, which are cleaved into sixteen NSPs including RdRp, 3C-like protease (3CLpro), papain-like protease (PLpro), and the helicase [53]. The replication process is carried out by the RdRp, also known as NSP12, in complex with NSP7 and NSP8 as cofactors that increase the polymerase activity [63,64]. Like all other polymerases, the overall structure of SARS-CoV-2 RdRp resembles a closed right hand with the palm, thumb, and finger subdomains. On the N-terminal tail, it has a nidovirus-specific domain with nucleotidyltransferase activity [65]. Unfortunately, only a few crystallographic structures of SARS-CoV RdRp have been solved to date, whereas no structures of MERS-CoV RdRp are available (Figure 6). In contrast, detailed information on *apo* RdRp structure, as well as the elucidation of the conformational changes of the protein by binding to RNA and a nucleoside analogue inhibitor, has been recently provided by Gao et al. (2020) [2] and Wang et al. (2020) [66]. In less than a year from the SARS-CoV-2 outbreak, nine three-dimensional structures of its RdRp were solved by Cryo-EM technique. 

### 4.2. Structure of Flaviviruses RdRp

The Flaviviruses’ positive single-stranded RNA has a single open reading frame (ORF) encoding a single polyprotein, which is later processed in three structural proteins (C-prM-E) and seven nonstructural proteins (NS1, NS2A, NS2B, NS3, NS4A, NS4B, and NS5) [67,68]. Flavivirus RdRp, also known as NS5, shows the classical “closed-hand” structure with palm–thumb–fingers subdomain. From a functional standpoint, RdRp initiates the replication process using the de novo initiation RNA synthesis mechanism. Unlike RdRps that use a primer-dependent mechanism such as RdRps of Picornaviruses and Caliciviruses, de novo RdRps have their own primer element often located in the thumb domain, which guides the 3’ tail of the RNA template strand within the catalytic site allowing the initiation of the complementary RNA strand synthesis [69,70]. Among viral proteins encoded by Flaviviruses, RdRp is the largest having around 900 amino acids [71,72] and consisting of two domains, i.e., the RdRp catalytic domain and the N-terminal MTase domain, that closely collaborate in the genome replication process. At the time of this review, 44 three-dimensional structures characterized by X-Ray crystallography are available in the Protein Data Bank (PDB) [73] for the Flaviviruses considered in this work: fourteen for ZIKV, three for WNV, and twenty-seven for DENV (Figure 6). For ZIKV and WNV, all the reported structures are in the *apo* conformation, whereas sixteen out of the twenty-seven DENV structures are co-crystallized with an allosteric inhibitor. Unfortunately, these structures only report the closed and catalytically non-competent conformation with no co-crystallized Mg^2+^ ions and RNA strands. Although sequence identity is not very high between Flaviviruses and HCV, X-Ray structures of HCV RdRp co-crystallized with a ligand, RNA, and Mg^2+^ ions allowed to generate reliable catalytically-competent structures of Flaviviruses’ RdRp through homology modeling [68,74].

### 4.3. Structure of HCV RdRp

HCV RdRp follows a structural pattern that is very similar to that of Flaviviruses belonging to the *Flaviviridae* family. A search in the PDB yielded 198 three-dimensional structures of HCV RdRp, corresponding to the 78% of total structures considered herein, all of which have been solved by X-Ray crystallography. Among them, three structures are co-crystallized with a nucleoside inhibitor, 160 with a non-nucleoside inhibitor, and fourteen are co-crystallized with double-stranded RNA and Mg^2+^ ions. It is worth noting that Mg^2+^ ions in some structures have been replaced by manganese ions (Mn^2+^) since they decrease the Michaelis constant (*K*_m_) and increase the polymerase activity of truncated RdRp even by 20-folds compared to Mg^2+^ ions, either with natural and 2’-OH/2’-CH_3_ modified ribonucleotide substrates [75,76] (Figure 6).

From these statistics, the strong interest in HCV drug discovery clearly emerges, which led to large investments in structural elucidation with appreciable results. Similarly, the strong engagement shown by the scientific community in the fight against the new coronavirus has demonstrated that it is possible to obtain remarkable results even in a relatively short time. Instead, in the case of Flaviviruses, this interest is apparently missing, suggesting a lower amount of resources spent so far.

## 5. Strategies for the Development of Small Molecule RdRp Inhibitors

Several strategies for the design of small molecule modulators of RNA viruses RdRp have been implemented, which have led to the development of multiple types of inhibitors endowed with different mechanisms of action: (i) nucleos(t)ide analogues (NIs), (ii) non-nucleos(t)ide analogue (NNIs), (iii) protein-protein interactions inhibitors, and (iv) targeted covalent inhibitors (TCIs).

**Figure 7:**
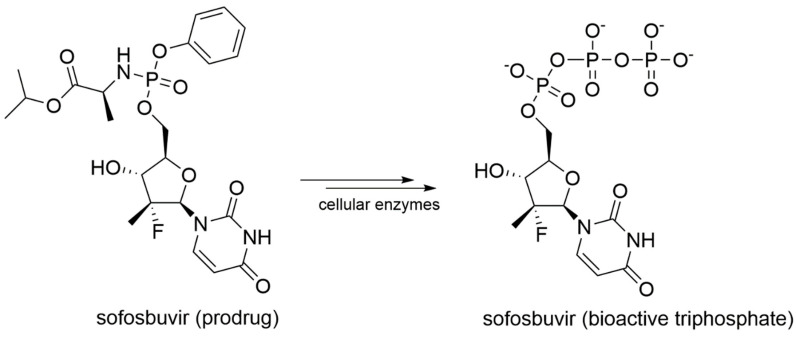
Schematic representation of the enzymatic transformation of sofosbuvir prodrug into its bioactive form.

In details, NIs are molecules that mimic the structure of nucleos(t)ides and act within the catalytic site of viral RdRp by binding metal ions and catalytic residues. They can be incorporated in the nascent RNA strand by competing with natural NTP substrates of the RdRp, leading to immediate or delayed chain termination depending on the chemical features of the small molecule inhibitor. The main limitation of NIs is their intracellular delivery in the form of triphosphates (bioactive form). Indeed, these moieties have a low cell membrane permeability due to the charge localized on the phosphate groups in physiological conditions. To overcome this problem, the prodrug approach is often used to improve the pharmacokinetic properties of a drug [77]. Prodrugs are pharmacologically inactive compounds that undergo enzymatic transformation(s) within the cell to become pharmacologically-active species. In the specific case of NIs, prodrugs are transformed and activated by cellular enzymes through one or more phosphorylation steps, allowing incorporation of the bioactive triphosphate into the nascent RNA strand with a catalytic mechanism that resembles the physiological process shown in Figure 2. A successful example of prodrug inhibitor of viral RdRp is sofosbuvir. Once metabolized in the bioactive compound GS-461203 (2’-deoxy-2’-α-fluoro-β-C-methyluridine-5’-triphosphate) (Figure 7), it acts as an obligated chain terminator. Although obligated chain terminators usually lack the 3’-hydroxyl group required for chain elongation, sofosbuvir has the 3’-hydroxyl group, but chain termination occurs due to the steric clashes of the 2’-methyl group [78].

Differently, NNIs are non-competitive inhibitors that bind allosteric sites of the polymerase, including thumb pockets I (T1) and II (T2), palm pockets I (P1), II (P2) and β (Pβ), which are adjacent to the active site, inhibiting the conformational changes required for the polymerase activity. The mechanism of action of allosteric inhibitors is highly dependent on the binding site and the conformational state of the polymerase [79]. NNIs binding to T1 and T2 seem to act at the initiation step by interfering with the correct assembly of the palm and thumb subdomains, thus preventing the formation of a functional RdRp/RNA complex and decreasing the binding affinity of the RNA template [80]. Palm site inhibitors have been shown to bind a hydrophobic pocket in the palm domain, stabilizing the inactive conformational states [81] or inhibiting the phosphodiester-bond formation [82]. Structural details on these sites are essential in the design of drugs that inhibit the RdRp through allosteric mechanisms.

A most recent class of RdRp inhibitors under development is represented by inhibitors of protein–protein interactions (PPI). In this case, RdRp inhibition occurs on the replication complex assembly between RdRp and other nonstructural proteins that exert enzymatic functions essential for the replication process. An example of PPI inhibition is the blockage of the interaction between RdRp (NS5) and the protease (NS3) of DENV [83], which has been demonstrated to be crucial for the replication of the viral genome by immunoprecipitation assay with cultured cells [84,85] and yeast two-hybrid assay [86].

TCIs are specifically designed with a functional group that is able to react with a specific residue of the target protein, forming a covalent complex that may lead to the loss of protein activity [87,88]. TCIs can be divided into two major classes by their mechanism of action: (i) reversible inhibitors, oscillating in an equilibrium between their bound and unbound state to the target. The recovery of the enzyme depends on the removal of the inhibitor from the system. (ii) Irreversible inhibitors that irreversibly binds the protein. In this case, the recovery of enzymatic functions does occur with the physiological turn-over [89], and their ability to closely tie their target results in a prolonged therapeutic response with a consequent decrease in doses and frequency of administration, and a significantly increased patient compliance [90]. Obviously, they also have disadvantages, such as episodes of toxicity and hypersensitivity [87,91]. In the development of TCIs, the target’s features should be thoroughly examined because not all the target proteins are suitable for this type of inhibition mechanism due to the high turn-over or degradation rate [92].

Recently, drug repurposing/repositioning has become a successful and established approach to rapidly enable compound development in advanced clinical trials and to accelerate the regulatory review process. Drug repurposing/repositioning aims to identify new therapeutic indications, different from the original medical indications, for approved, investigational, or withdrawn/suspended drugs [93]. This strategy offers several advantages over the canonical process to develop a new drug. Since pharmacokinetic, toxicological, and safety data have already been collected for the repurposed drug in preclinical and early clinical trials, the risk of failure is considerably lower. Then, fewer investments are required, although depending on the phase and process of development achieved by the candidate drug [94]. There are many successful examples of drug repurposing/repositioning, including sildenafil, originally designed to treat angina pectoris, to date used for the treatment of erectile dysfunction [95]. Recently, drug repurposing proved very beneficial in emergency situations, such as the COVID-19 pandemic [96,97,98], which led to the approval of remdesivir, an RdRp inhibitor originally developed against HCV and later proposed for the treatment of Ebola virus infection. This approach is also being used for the treatment of Flavivirus infections [74,78,99] and drugs under repurposing for the treatment of Flavivirus infections are mostly anti-HCV agents.

It is worth noting that inhibition of RdRp activity through modulation of structural motifs or viral nucleic acid sequences targeted by the RdRp stands as a viable option for the development of antiviral agents [100]. Specifically, peptide nucleic acid (PNA) oligomers are artificial nucleic acids with the nucleobases anchored to a neutral pseudopeptide backbone via a methylene carbonyl linkage [101]. PNAs are reported to bind selectively to specific viral RNA secondary structures providing indirect inhibition of RdRp.

In the next chapters, successful examples of Coronavirus, Flavivirus, and HCV RdRp inhibitors are described. Since there are several approved and investigational RdRp inhibitors for HCV treatment, only those commercially available and in advanced clinical trials having a representative mechanism of action will be reported.

### 5.1. Small Molecule Inhibitors of Coronaviruses’ RdRp

**Figure 8:**
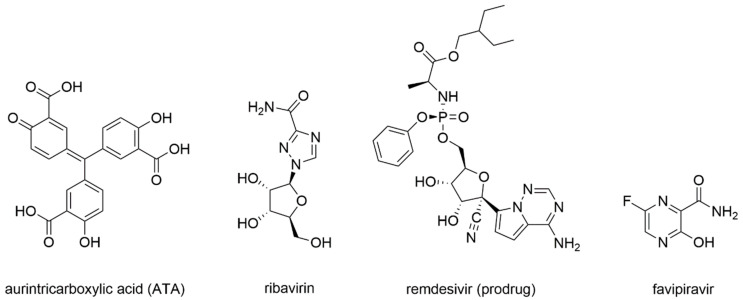
Small molecule inhibitors of the RdRp of Coronaviruses.

First studies on the inhibition of Coronaviruses’ RdRp were carried out in 2004, following the SARS-CoV epidemic. As already summarized in the work of Wu et al. (2006) [102], cell-based studies identified aurintricarboxylic acid (ATA) (Figure 8) as a selective inhibitor of SARS-CoV replication with promising results. Indeed, viral replication was decreased around 1000-fold with respect to the untreated control, and even when compared with interferon α and β, ATA resulted in 10 and 100 times more potency, respectively [103].

Then, docking studies were carried out to investigate the potential binding mode of ATA onto SARS-CoV RdRp, confirming that it was able to bind in a pocket located in the palm subdomain, formed by S754–Y766 residues, which also includes two aspartate residues involved in binding the metal ions that participate in the catalytic process [104].

Later studies, reviewed in Totura et al. (2019) [105], reported that the guanosine analogue ribavirin (Figure 8) was also tested against SARS-CoV and MERS-CoV infections. SARS-CoV and MERS-CoV were shown to be sensitive to ribavirin alone in cell-based assays, but at high concentrations difficult to achieve in human serum. Combinations with interferons were tried and led to similar results with lower ribavirin concentrations [106,107]. Given these results, combinations of ribavirin and interferon-α2b were tested in vivo on primate animal models improving the clinical outcome of early infections [108]. Unfortunately, this treatment did not provide benefits to patients with severe respiratory pathology. Moreover, once incorporated in the nascent RNA strand, ribavirin was subject to cellular excision mechanisms from the NSP14 exoribonuclease, a bifunctional enzyme with MTase activity that is able to excise erroneous mutagenic nucleotides inserted by the RdRp, which could explain the limited drug activity in vivo [109].

**Figure 9:**
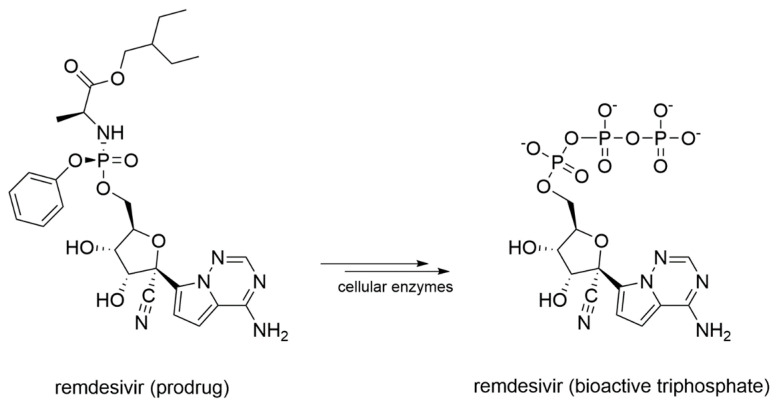
Schematic representation of the enzymatic transformation of remdesivir prodrug into its bioactive form.

Remdesivir, initially designed as an anti-HCV drug, was then screened from a library of ~1000 compounds as a potential treatment option for Ebola virus and Marburg virus during the outbreak of 2014 in West Africa [110]. Since it proved to be a broad-spectrum antiviral agent, it was also tested against MERS-CoV, showing an IC_50_ of 340 nM in in-vitro cell-based assays [111]. Remdesivir activity against SARS-CoV and MERS-CoV was also assessed in multiple in vitro systems providing IC_50_ values in the sub-micromolar range [112]. Later, it was found to have prophylactic and therapeutic activity in a primate model of MERS-CoV infection [113]. From a chemical standpoint, remdesivir is a phosphoramidate prodrug (Figure 9) of an adenine derivative with broad-spectrum antiviral activity, which acts as a delayed chain terminator. 

The presence of the 3’-hydroxyl group still allows the formation of the phosphodiester bond and the elongation of the growing RNA chain. Only after the addition of three other NTPs, the steric hindrance caused by the 1’-CN group with the S861 residue blocks the transcription/replication process [114]. Moreover, in SARS-CoV and MERS-CoV, the F480L/V557L recombinant mutant in the finger domain conferred up to 6-fold resistance to remdesivir [115]. Since previous outbreaks of SARS-CoV and MERS-CoV were much shorter and more restricted compared to the current SARS-CoV-2 pandemic, most antiviral development programs were discontinued, and for the diseases caused by these Coronaviruses, no specific treatment has been approved [116]. However, the high percentage of sequence identity between SARS-CoV and SARS-CoV-2 RdRp prompted an assessment of remdesivir as a COVID-19 medication [117]. Moreover, the Cryo-EM structure of remdesivir within the catalytic site of SARS-CoV-2 RdRp provided structural insights into its mechanism of action, and became a profitable rational template for the development of novel small molecule inhibitors of RdRp (PDB-ID: 7BV2) [63]. Remdesivir was indeed approved for the treatment of hospitalized patients with severe COVID-19 [118,119]. Nevertheless, the demand for effective anti-SARS-CoV-2 drugs remains high.

**Figure 10:**
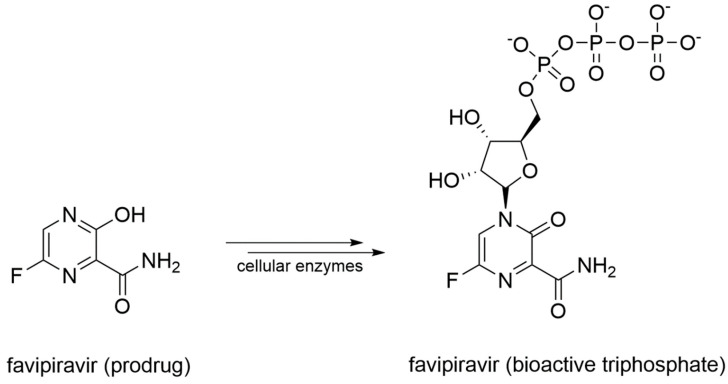
Schematic representation of the enzymatic transformation of favipiravir prodrug into its bioactive form.

Favipiravir is a prodrug that undergoes a metabolic transformation into favipiravir triphosphate, its bioactive form (Figure 10).

**Figure 11:**
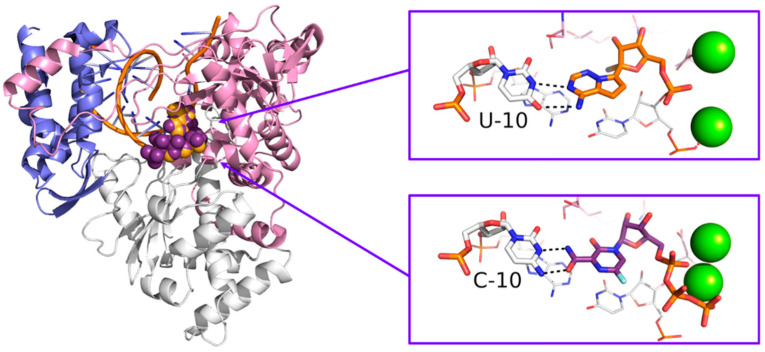
Overview of the binding mode of remdesivir (orange PDB-ID: 7BV2), and favipiravir (violet PDB-ID: 7AAP), within the catalytic site of RdRp of SARS-CoV-2. Residues opposed to the drugs are represented as sticks and are labeled. Base-base contacts are highlighted by black dashed lines.

Originally designed as an anti-influenza drug and currently approved with this indication in Japan, favipiravir acts as a false substrate for SARS-CoV-2 RdRp with a combined mechanism of lethal mutagenesis and chain termination [120]. Two crystallographic structures of favipiravir within the catalytic site of RdRp of SARS-CoV-2 are available, which describe the pre-catalytic and the catalytically-competent state of the protein with Mg^2+^ ions and double-stranded RNA, helping to clarify the drug’s mechanism of action at a molecular level (PDB-ID: 7CTT, 7AAP). Favipiravir was used to treat mild and moderate COVID-19 in several countries based on emergency approval. Clinical studies to assess its efficacy in the treatment of COVID-19 alone or in combination with other drugs are ongoing [121]. An overview of remdesivir and favipiravir within the catalytic site of the RdRp of SARS-CoV-2 is depicted in Figure 11.

### 5.2. Small Molecule Inhibitors of Flaviviruses’ RdRp

**Figure 12:**
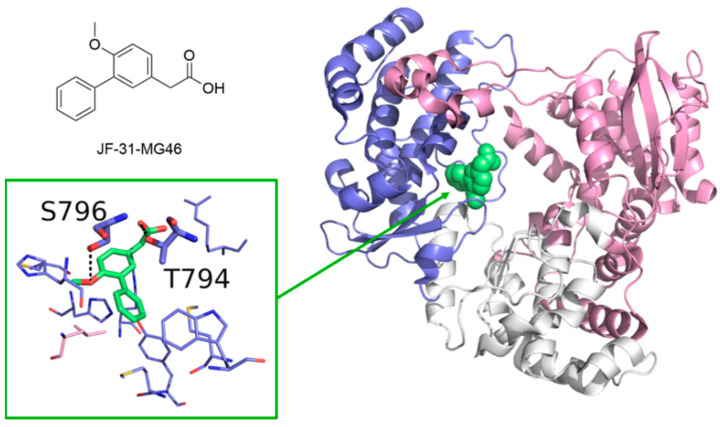
Crystallographic structure of DENV in complex with a small molecule inhibitor bound into the N-pocket site (PDB-ID: 5F3T). Residues involved in polar interactions are represented as sticks and are labeled. Polar contacts are highlighted by black dashed lines.

No drugs for the treatment of Flavivirus infections have been approved to date. On one hand, this may be due the limited interest and research efforts towards these viruses, for example, compared to Coronavirus and HCV. On the other hand, the lack of structural information on the catalytically-competent RdRp in complex with RNA strands and metal ions notably hampers the design of new small molecule inhibitors. Recently, many studies focused on repositioning the nucleoside analogue sofosbuvir (Figure 7), approved in 2013 by the US Food and Drug Administration (FDA) for the treatment of chronic HCV infection [122], as a lead candidate against ZIKV, DENV, and WNV. The drug was tested against ZIKV on different cellular systems and shown to directly inhibit ZIKV replication, acting both as a chain terminator and inducing A-to-G mutation in the viral genome [99]. Sofosbuvir also inhibited DENV replication in both cell-based and biochemical assays [78] and demonstrated anti-WNV activity on purified RdRP with an IC_50_ value of 11.1 μM [74]. Moreover, the S604T mutation in WNV RdRp confers sofosbuvir resistance, corresponding to the S282T in HCV [74]. Following the identification of several allosteric sites in HCV RdRp, a number of studies have focused on the development of inhibitors of Flavivirus RdRp. An HCV P1-like site has been identified in DENV RdRp using fragment-based screening via X-Ray crystallography, and it is known as N-pocket (Figure 12) [123,124].

The same pocket was identified and targeted for inhibition of ZIKV RdRp. The most potent compound tested so far on ZIKA RdRp had an IC_50_ value of 7.3 μM in cell-free assays and showed inhibition of ZIKV replication in vitro with an EC_50_ value of 24.3 μM. However, crystallographic structures of four compounds binding the RdRp in the N-pocket helped to clarify their binding mode and highlighted the difference between the RdRp of ZIKV and DENV, information that may become essential for the design of specific small molecule inhibitors [125]. In HCV, DENV, and ZIKV RdRps, this site is located at the palm/thumb subdomains interface, and it appears to be a promising target for broad-spectrum antivirals [126]. Recent studies on DENV also highlighted the importance of the NS5-NS3 PPI for the correct assembly of the replication complex and demonstrated that inhibition of these PPIs could be a profitable strategy to impair the replication transduction pathway and to develop novel broad-spectrum antiviral therapeutic agents [127].

### 5.3. Small Molecules Inhibitors of HCV RdRp

Until a few years ago, the treatment of Hepatitis C was based on the combination of ribavirin and PEG-interferon-α. Depending on HCV genotype, effectiveness was 40–75%, but many patients were not eligible for this treatment, and side effects such as cardiovascular, neurological, or psychiatric disorders were common [128]. Successful therapy is defined by the undetectability of serum HCV-RNA twelve weeks after cessation of treatment, a condition referred to as sustained virologic response (SVR) [129]. In 2011, the first HCV protease inhibitors, boceprevir and telaprevir, were disclosed. These compounds form a reversible covalent bond with the S139 from the catalytic triad of the HCV NS3 protease (S139, H57, and D81), inhibiting its catalytic activity [130]. The discovery of these compounds shed light on the importance of identifying compounds that directly target viral proteins [131]. These kinds of molecules are defined as directly acting antivirals (DAA), due to the direct activity on viral proteins, as opposed to the previous interferon-based standard of care [132]. A number of potent DAAs were later developed, which are the basis for current, pan-genotypic, interferon-free, well-tolerated, and highly effective HCV treatment regimens [133,134]. 

#### 5.3.1. RdRp Inhibitors Approved for the Treatment of HCV Infection

##### Sofosbuvir

**Figure 13:**
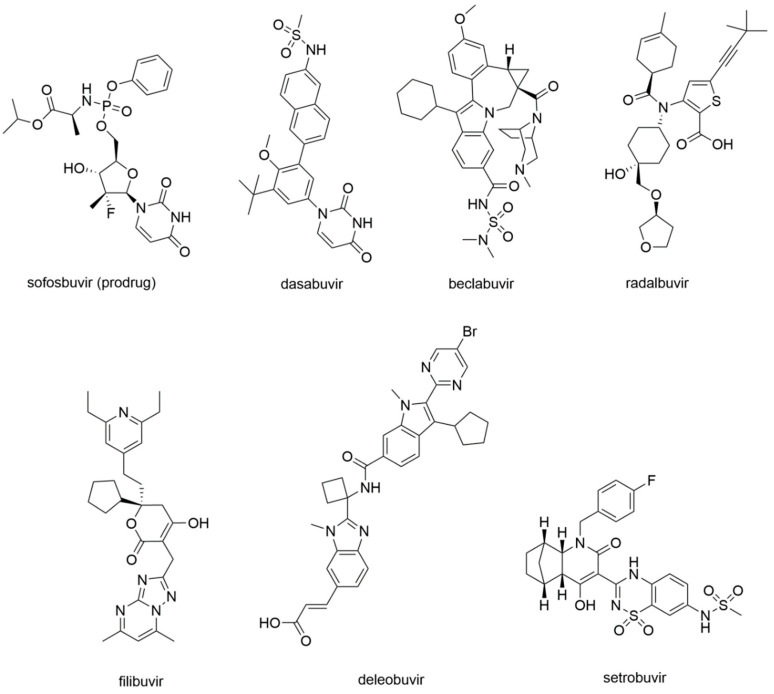
Small molecule inhibitors of the RdRp of HCV.

In 2013, sofosbuvir (Figure 13) was approved by the FDA for the treatment of chronic HCV infection as a RdRp nucleoside analogue inhibitor.

**Figure 14:**
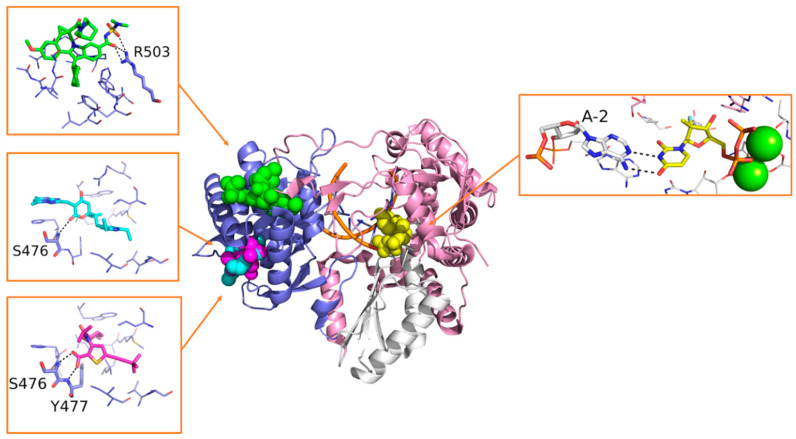
Overview of compounds sofosbuvir (yellow, PDB-ID: 4WTG), beclabuvir (green, PDB-ID: 4NLD), radalbuvir (magenta, PDB-ID: 4EO6), and filibuvir (cyan, PDB-ID: 3FRZ) co-crystallized into the different binding sites of the RdRp of HCV. Residues involved in polar interactions are represented as sticks and are labeled. Polar contacts are highlighted by black dashed lines.

Sofosbuvir is synthesized as a prodrug, and once metabolized in its bioactive form 2’-deoxy-2’-α-fluoro-β-C-methyluridine-5’-monophosphate (Figure 7), it is incorporated into the nascent RNA, mimicking the UTP and causing chain termination. The S282T mutation within the catalytic site of HCV RdRp confers a modest level of resistance to sofosbuvir in vivo (3× to 10× fold-change in median effective concentration [EC_50_]), and it is unfit for viral replication (replication fitness approximately 8% of WT) [135]. Sofosbuvir was also successfully co-crystallized within the catalytic site of HCV RdRp, with double-stranded RNA and Mn^2+^ ions (PDB-ID: 4WTG) [75]. The crystallographic study provided more detailed information on the mechanism of action of sofosbuvir and on the interactions established with the protein. Structural details on the binding mode of sofosbuvir and other small molecule inhibitors co-crystallized with HCV RdRp are provided in Figure 14.

##### Dasabuvir

In 2014, the RdRp NNI dasabuvir (Figure 13) was approved by the FDA for the treatment of HCV infection with the ombitasvir–paritaprevir–ritonavir association. Dasabuvir is an aryl dihydrouracil derivate that was identified from a high-throughput screening campaign of the aryl dihydrouracil fragment [136]. It is a P1 inhibitor that binds a hydrophobic pocket adjacent to the active site, blocking the RNA chain initiation steps of the RdRp replication process with an allosteric mechanism [137]. 

#### 5.3.2. Investigational and Discontinued HCV RdRp Inhibitors

The impressive success of current HCV DAA regimens has led to premature termination of most drug development programs in this area, although some novel drugs are still being explored. As a result, several promising compounds were discontinued, which could be repositioned in neighbor areas such as other viral RdRps. Since structural information is sometimes available, past candidate HCV RdRp inhibitors may play a key role in driving a structure-based approach.

##### Beclabuvir

Beclabuvir (Figure 13) is an NNI indole derivate that binds to the T1 site as depicted by X-Ray crystallography (Figure 14). In this case, structural studies proved crucial to confirm the compound’s preferred stereochemistry at the ring junction for optimal antiviral activity, as well as to provide molecular details of its mechanism of action (PDB-ID: 4NLD) [138]. A phase 3 clinical study on beclabuvir is running, in combination with daclatasvir and asunaprevir (NCT02170727), whereas a safety surveillance study on the same combination of drugs is running in Japan (NCT03071133).

##### Radalbuvir

Radalbuvir (Figure 13) is also an NNI currently in phase 2 clinical stage, acting at the T2 site of HCV RdRp as elucidated by X-Ray crystallography (Figure 14). In this case, structural information emerged as a reliable tool in the optimization of thiophene-2-carboxylic acid inhibitors of HCV RdRp (PDB-ID: 4EO6) [139].

##### Filibuvir

Filibuvir (Figure 13) is an RdRp inhibitor that binds the T2 site. Its discovery was guided by X-Ray crystallography (Figure 14), starting from the class of dihydropyranones (PDB-ID: 3FRZ). Although filibuvir was shown to be a potent viral replication inhibitor with a mean IC_50_ of 0.007 μM [140], the genetic barrier to resistance is low [141] and similar to other NNIs. Therefore, its development was discontinued due to strategic reasons [142]. 

##### Deleobuvir and Setrobuvir

Deleobuvir and setrobuvir (Figure 13) are both NNI of the HCV RdRp. Deleobuvir reversibly and non-covalently binds to the T1 site [143], while setrobuvir binds the P1 allosteric site of the enzyme [144]. Their development process was terminated due to the lack of efficacy that emerged in clinical studies.

## 6. Conclusions

The approval of sofosbuvir and remdesivir as specific RdRp inhibitors for the treatment of HCV and SARS-CoV-2 infections, respectively, marked a fundamental step in antiviral drug discovery. In contrast, no drugs have been licensed against other RNA viruses coding for an RdRp, for example, Flaviviruses. The success obtained so far with inhibitors of the RdRp from only two of the many viruses of this kind probably reflects the disproportionate effort devoted to these agents compared to others. The HCV RdRp is undoubtedly the most studied among viral RdRps, due to the high morbidity and mortality of HCV infection coupled with lack of vaccination. The recent SARS-CoV-2 pandemic is an unprecedented emergency, strongly supporting drug repurposing as an immediate, at least partially active strategy while waiting for specific drug development. 

Based on the evidence presented in this review, it is highly plausible that drugs acting on the RdRp of Flaviviruses might be developed with success if multidisciplinary and concerted efforts are dedicated to this task. It is also expected that these drugs will enhance the preparedness of health systems and international organizations for possible future pandemics. In this respect, the lack of structural details on catalytically-competent or ligand-bound RdRps from Flaviriruses marks the difference with HCV and SARS-CoV-2, and might limit the successful application of structure-based drug design either in the repositioning and conventional approaches. 

Overall, in this review, we summarized the recent findings in targeting the RdRp of RNA viruses. The success stories discussed herein, together with structural hints, should inspire the design of additional drug candidates, and set the basis for expanding structure-based approaches.

## References

[1] Černý J., Bolfíková B.Č., Valdés J.J., Grubhoffer L., Růžek D. (2014). Evolution of Tertiary Structure of Viral RNA Dependent Polymerases. PLoS ONE.

[2] Gao Y., Yan L., Huang Y., Liu F., Zhao Y., Cao L., Wang T., Sun Q., Ming Z., Zhang L. (2020). Structure of the RNA-dependent RNA polymerase from COVID-19 virus. Science.

[3] Malet H., Massé N., Selisko B., Romette J.-L., Alvarez K., Guillemot J.C., Tolou H., Yap T.L., Vasudevan S.G., Lescar J. (2008). The flavivirus polymerase as a target for drug discovery. Antivir. Res..

[4] Zhao Y., Soh T.S., Zheng J., Chan K.W.K., Phoo W.W., Lee C.C., Tay M.Y.F., Swaminathan K., Cornvik T.C., Lim S.P. (2015). A Crystal Structure of the Dengue Virus NS5 Protein Reveals a Novel Inter-domain Interface Essential for Protein Flexibility and Virus Replication. PLoS Pathog..

[5] Carvalho A.T.P., Fernandes P.A., Ramos M.J. (2011). The Catalytic Mechanism of RNA Polymerase II. J. Chem. Theory Comput..

[6] Buck K.W. (1996). Comparison of the Replication of Positive-Stranded Rna Viruses of Plants and Animals. Adv. Virus Res..

[7] Siegel R.W., Bellon L., Beigelman L., Kao C.C. (1998). Moieties in an RNA promoter specifically recognized by a viral RNA-dependent RNA polymerase. Proc. Natl. Acad. Sci. USA.

[8] Peck K.M., Lauring A.S. (2018). Complexities of Viral Mutation Rates. J. Virol..

[9] Shaevitz J.W., Abbondanzieri E.A., Landick R., Block S.M. (2003). Backtracking by single RNA polymerase molecules observed at near-base-pair resolution. Nature.

[10] Dulin D., Vilfan I.D., Berghuis B.A., Poranen M.M., Depken M., Dekker N.H. (2015). Backtracking behavior in viral RNA-dependent RNA polymerase provides the basis for a second initiation site. Nucleic Acids Res..

[11] Robson F., Khan K.S., Le T.K., Paris C., Demirbag S., Barfuss P., Rocchi P., Ng W.-L. (2020). Coronavirus RNA Proofreading: Molecular Basis and Therapeutic Targeting. Mol. Cell.

[12] de Farias S.T., dos Santos Junior A.P., Rêgo T.G., José M.V. (2017). Origin and Evolution of RNA-Dependent RNA Polymerase. Front. Genet..

[13] Chan-yeung M., Xu R. (2003). SARS: Epidemiology. Respirology.

[14] Nassar M.S., Bakhrebah M.A., Meo S.A., Alsuabeyl M.S., Zaher W.A. (2018). Middle East Respiratory Syndrome Coronavirus (MERS-CoV) infection: Epidemiology, pathogenesis and clinical characteristics. Eur. Rev..

[15] Mori M., Capasso C., Carta F., Donald W.A., Supuran C.T. (2020). A deadly spillover: SARS-CoV-2 outbreak. Expert Opin. Ther. Pat..

[16] de Paula Freitas B., Ventura C.V., Maia M., Belfort R.J. (2017). Zika virus and the eye. Curr. Opin. Ophthalmol..

[17] Medin C.L., Rothman A.L. (2017). Zika Virus: The Agent and Its Biology, With Relevance to Pathology. Arch. Pathol. Lab. Med..

[18] Dick G.W.A., Kitchen S.F., Haddow A.J. (1952). Zika Virus (I). Isolations and serological specificity. Trans R. Soc. Trop. Med. Hyg..

[19] de Oliveira Dias J.R., Ventura C.V., de Paula Freitas B., Prazeres J., Ventura L.O., Bravo-Filho V., Aleman T., Ko A.I., Zin A., Belfort R. (2018). Zika and the Eye: Pieces of a Puzzle. Prog. Retin. Eye Res..

[20] Weaver S.C., Costa F., Garcia-Blanco M.A., Ko A.I., Ribeiro G.S., Saade G., Shi P.-Y., Vasilakis N. (2016). Zika virus: History, emergence, biology, and prospects for control. Antivir. Res..

[21] Salehuddin A.R., Haslan H., Mamikutty N., Zaidun N.H., Azmi M.F., Senin M.M., Syed Ahmad Fuad S.B., Thent Z.C. (2017). Zika virus infection and its emerging trends in Southeast Asia. Asian Pac. J. Trop. Med..

[22] Wikan N., Smith D.R. (2016). Zika virus: History of a newly emerging arbovirus. Lancet Infect. Dis..

[23] Carlson C.J., Dougherty E.R., Getz W. (2016). An Ecological Assessment of the Pandemic Threat of Zika Virus. PLoS Negl. Trop. Dis..

[24] Gyawali N., Bradbury R.S., Taylor-Robinson A.W. (2016). The global spread of Zika virus: Is public and media concern justified in regions currently unaffected?. Infect. Dis. Poverty.

[25] Jaenisch T., Rosenberger K.D., Brito C., Brady O., Brasil P., Marques E.T. (2017). Risk of microcephaly after Zika virus infection in Brazil, 2015 to 2016. Bull. World Health Organ..

[26] Brasil P., Pereira J.P., Moreira M.E., Nogueira R.M.R., Damasceno L., Wakimoto M., Rabello R.S., Valderramos S.G., Halai U.-A., Salles T.S. (2016). Zika Virus Infection in Pregnant Women in Rio de Janeiro. N. Engl. J. Med..

[27] Harapan H., Michie A., Sasmono R.T., Imrie A. (2020). Dengue: A Minireview. Viruses.

[28] Messina J.P., Brady O.J., Scott T.W., Zou C., Pigott D.M., Duda K.A., Bhatt S., Katzelnick L., Howes R.E., Battle K.E. (2014). Global spread of dengue virus types: Mapping the 70 year history. Trends Microbiol..

[29] Hotta S. (1952). Experimental Studies on DengueI. Isolation, Identification and Modification of the Virus. J. Infect. Dis..

[30] Gubler D.J. (2011). Dengue, Urbanization and Globalization: The Unholy Trinity of the 21st Century. Trop. Med. Health.

[31] Schaffner F., Mathis A. (2014). Dengue and dengue vectors in the WHO European region: Past, present, and scenarios for the future. Lancet Infect. Dis..

[32] Zaayman D., Venter M. (2012). West Nile Virus Neurologic Disease in Humans, South Africa, September 2008–May 2009-Volume 18, Number 12—December. Emerg. Infect. Dis. J. CDC.

[33] Popescu C.P., Florescu S.A., Cotar A.I., Badescu D., Ceianu C.S., Zaharia M., Tardei G., Codreanu D., Ceausu E., Ruta S.M. (2018). Re-emergence of severe West Nile virus neuroinvasive disease in humans in Romania, 2012 to 2017–implications for travel medicine. Travel Med. Infect. Dis..

[34] Magurano F., Remoli M.E., Baggieri M., Fortuna C., Marchi A., Fiorentini C., Bucci P., Benedetti E., Ciufolini M.G., Rizzo C. (2012). Circulation of West Nile virus lineage 1 and 2 during an outbreak in Italy. Clin. Microbiol. Infect..

[35] Nash D., Mostashari F., Fine A., Miller J., O’Leary D., Murray K., Huang A., Rosenberg A., Greenberg A., Sherman M. (2001). The Outbreak of West Nile Virus Infect. in the New York City Area in 1999. N. Engl. J. Med..

[36] Lanciotti R.S., Roehrig J.T., Deubel V., Smith J., Parker M., Steele K., Crise B., Volpe K.E., Crabtree M.B., Scherret J.H. (1999). Origin of the West Nile Virus Responsible for an Outbreak of Encephalitis in the Northeastern United States. Science.

[37] Frost M.J., Zhang J., Edmonds J.H., Prow N.A., Gu X., Davis R., Hornitzky C., Arzey K.E., Finlaison D., Hick P. (2012). Characterization of Virulent West Nile Virus Kunjin Strain, Australia, 2011-Volume 18, Number 5—May. Emerg. Infect. Dis. J. CDC.

[38] Musso D., Nilles E.J., Cao-Lormeau V.-M. (2014). Rapid spread of emerging Zika virus in the Pacific area. Clin. Microbiol. Infect..

[39] Petruzziello A., Marigliano S., Loquercio G., Cozzolino A., Cacciapuoti C. (2016). Global epidemiology of hepatitis C virus infection: An up-date of the distribution and circulation of hepatitis C virus genotypes. World J. Gastroenterol..

[40] Sharma S.D. (2010). Hepatitis C virus: Molecular biology & current therapeutic options. Indian J. Med. Res..

[41] Maheshwari A., Thuluvath P.J. (2010). Management of Acute Hepatitis C. Clin. Liver Dis..

[42] Zelenev A., Jianghong L., Mazhnaya A., Basu S., Altice F.L. (2018). Hepatitis C virus treatment as prevention in an extended network of people who inject drugs in the USA: A modelling study. Lancet Infect. Dis..

[43] Janiak M., Caraballo Cortes K., Demkow U., Radkowski M., Pokorski M. (2018). Spontaneous Elimination of Hepatitis C Virus Infection. Current Concepts in Medical Research and Practice.

[44] Rosen H. (2011). Clinical practice. Chronic hepatitis C infection. N. Engl. J. Med..

[45] Gorbalenya A.E., Baker S.C., Baric R.S., de Groot R.J., Drosten C., Gulyaeva A.A., Haagmans B.L., Lauber C., Leontovich A.M., Neuman B.W. (2020). The species severe acute respiratory syndrome-related coronavirus: Classifying 2019-nCoV and naming it SARS-CoV-2. Nat. Microbiol..

[46] Shrivastava S., Puri V., Dilley K.A., Ngouajio E., Shifflett J., Oldfield L.M., Fedorova N.B., Hu L., Williams T., Durbin A. (2018). Whole genome sequencing, variant analysis, phylogenetics, and deep sequencing of Zika virus strains. Sci. Rep..

[47] Marra M.A., Jones S.J.M., Astell C.R., Holt R.A., Brooks-Wilson A., Butterfield Y.S.N., Khattra J., Asano J.K., Barber S.A., Chan S.Y. (2003). The Genome Sequence of the SARS-Associated Coronavirus. Science.

[48] Richter J., Tryfonos C., Tourvas A., Floridou D., Paphitou N.I., Christodoulou C. (2017). Complete Genome Sequence of West Nile Virus (WNV) from the First Human Case of Neuroinvasive WNV Infect. in Cyprus. Genome Announc..

[49] Kandeil A., Shehata M.M., El Shesheny R., Gomaa M.R., Ali M.A., Kayali G. (2016). Complete Genome Sequence of Middle East Respiratory Syndrome Coronavirus Isolated from a Dromedary Camel in Egypt. Genome Announc..

[50] Sah R., Rodriguez-Morales A.J., Jha R., Chu D.K.W., Gu H., Peiris M., Bastola A., Lal B.K., Ojha H.C., Rabaan A.A. (2020). Complete Genome Sequence of a 2019 Novel Coronavirus (SARS-CoV-2) Strain Isolated in Nepal. Microbiol. Resour. Announc..

[51] Dang T.T., Pham M.H., Bui H.V., Le D.V. (2020). Whole genome sequencing and genetic variations in several dengue virus type 1 strains from unusual dengue epidemic of 2017 in Vietnam. Virol. J..

[52] Pérez-Losada M., Arenas M., Galán J.C., Bracho M.A., Hillung J., García-González N., González-Candelas F. (2020). High-throughput sequencing (HTS) for the analysis of viral populations. Infect. Genet. Evol..

[53] Xu X., Liu Y., Weiss S., Arnold E., Sarafianos S.G., Ding J. (2003). Molecular model of SARS coronavirus polymerase: Implications for biochemical functions and drug design. Nucleic Acids Res..

[54] Larkin M.A., Blackshields G., Brown N.P., Chenna R., McGettigan P.A., McWilliam H., Valentin F., Wallace I.M., Wilm A., Lopez R. (2007). Clustal W and Clustal X version 2.0. Bioinformatics.

[55] Rost B. (1999). Twilight zone of protein sequence alignments. Protein Eng. Des. Sel..

[56] Lu G., Gong P. (2013). Crystal Structure of the Full-Length Japanese Encephalitis Virus NS5 Reveals a Conserved Methyltransferase-Polymerase Interface. PLoS Pathog..

[57] Thompson A.A., Peersen O.B. (2004). Structural basis for proteolysis-dependent activation of the poliovirus RNA-dependent RNA polymerase. EMBO J..

[58] Xu H.-T., Hassounah S.A., Colby-Germinario S.P., Oliveira M., Fogarty C., Quan Y., Han Y., Golubkov O., Ibanescu I., Brenner B. (2017). Purification of Zika virus RNA-dependent RNA polymerase and its use to identify small-molecule Zika inhibitors. J. Antimicrob. Chemother..

[59] Venkataraman S., Prasad B.V.L.S., Selvarajan R. (2018). RNA Dependent RNA Polymerases: Insights from Structure, Function and Evolution. Viruses.

[60] Ahmed-Belkacem A., Guichou J.-F., Brillet R., Ahnou N., Hernandez E., Pallier C., Pawlotsky J.-M. (2014). Inhibition of RNA binding to hepatitis C virus RNA-dependent RNA polymerase: A new mechanism for antiviral intervention. Nucleic Acids Res..

[61] Zhou Z., Liu T., Zhang J., Zhan P., Liu X. (2018). Influenza a virus polymerase: An attractive target for next-generation anti-influenza therapeutics. Drug Discov. Today.

[62] Ferrer-Orta C., Agudo R., Domingo E., Verdaguer N. (2009). Structural insights into replication initiation and elongation processes by the FMDV RNA-dependent RNA polymerase. Curr. Opin. Struct. Biol..

[63] Yin W., Mao C., Luan X., Shen D.-D., Shen Q., Su H., Wang X., Zhou F., Zhao W., Gao M. (2020). Structural basis for inhibition of the RNA-dependent RNA polymerase from SARS-CoV-2 by remdesivir. Science.

[64] Subissi L., Posthuma C.C., Collet A., Zevenhoven-Dobbe J.C., Gorbalenya A.E., Decroly E., Snijder E.J., Canard B., Imbert I. (2014). One severe acute respiratory syndrome coronavirus protein complex integrates processive RNA polymerase and exonuclease activities. Proc. Natl. Acad. Sci. USA.

[65] Lehmann K.C., Gulyaeva A., Zevenhoven-Dobbe J.C., Janssen G.M.C., Ruben M., Overkleeft H.S., van Veelen P.A., Samborskiy D.V., Kravchenko A.A., Leontovich A.M. (2015). Discovery of an essential nucleotidylating activity associated with a newly delineated conserved domain in the RNA polymerase-containing protein of all nidoviruses. Nucleic Acids Res..

[66] Wang Q., Wu J., Wang H., Gao Y., Liu Q., Mu A., Ji W., Yan L., Zhu Y., Zhu C. (2020). Structural Basis for RNA Replication by the SARS-CoV-2 Polymerase. Cell.

[67] Tham H.-W., Balasubramaniam V.R.M.T., Chew M.-F., Ahmad H., Hassan S.S. (2015). Protein-protein interactions between A. aegypti midgut and dengue virus 2: Two-hybrid screens using the midgut cDNA library. J. Infect. Dev. Ctries..

[68] Šebera J., Dubankova A., Sychrovský V., Ruzek D., Boura E., Nencka R. (2018). The structural model of Zika virus RNA-dependent RNA polymerase in complex with RNA for rational design of novel nucleotide inhibitors. Sci. Rep..

[69] Ferrer-Orta C., Arias A., Escarmís C., Verdaguer N. (2006). A comparison of viral RNA-dependent RNA polymerases. Curr. Opin. Struct. Biol..

[70] Butcher S.J., Grimes J.M., Makeyev E.V., Bamford D.H., Stuart D.I. (2001). A mechanism for initiating RNA-dependent RNA polymerization. Nature.

[71] Piorkowski G., Richard P., Baronti C., Gallian P., Charrel R., Leparc-Goffart I., de Lamballerie X. (2016). Complete coding sequence of Zika virus from Martinique outbreak in 2015. New Microbes New Infect..

[72] Issur M., Geiss B.J., Bougie I., Picard-Jean F., Despins S., Mayette J., Hobdey S.E., Bisaillon M. (2009). The flavivirus NS5 protein is a true RNA guanylyltransferase that catalyzes a two-step reaction to form the RNA cap structure. RNA.

[73] Berman H.M., Westbrook J., Feng Z., Gilliland G., Bhat T.N., Weissig H., Shindyalov I.N., Bourne P.E. (2000). The Protein Data Bank. Nucleic Acids Res..

[74] Dragoni F., Boccuto A., Picarazzi F., Giannini A., Giammarino F., Saladini F., Mori M., Mastrangelo E., Zazzi M., Vicenti I. (2020). Evaluation of sofosbuvir activity and resistance profile against West Nile virus in vitro. Antivir. Res..

[75] Appleby T.C., Perry J.K., Murakami E., Barauskas O., Feng J., Cho A., Fox D., Wetmore D.R., McGrath M.E., Ray A.S. (2015). Structural basis for RNA replication by the hepatitis C virus polymerase. Science.

[76] Luo G., Hamatake R.K., Mathis D.M., Racela J., Rigat K.L., Lemm J., Colonno R.J. (2000). De Novo Initiation of RNA Synthesis by the RNA-Dependent RNA Polymerase (NS5B) of Hepatitis C Virus. J. Virol..

[77] Slusarczyk M., Serpi M., Pertusati F. (2018). Phosphoramidates and phosphonamidates (ProTides) with antiviral activity. Antivir Chem. Chemother..

[78] Xu H.-T., Colby-Germinario S.P., Hassounah S.A., Fogarty C., Osman N., Palanisamy N., Han Y., Oliveira M., Quan Y., Wainberg M.A. (2017). Evaluation of Sofosbuvir (β-D-2′-deoxy-2′-α-fluoro-2′-β-C-methyluridine) as an inhibitor of Dengue virus replication. Sci. Rep..

[79] Eltahla A.A., Luciani F., White P.A., Lloyd A.R., Bull R.A. (2015). Inhibitors of the Hepatitis C Virus Polymerase; Mode of Action and Resistance. Viruses.

[80] Winquist J., Abdurakhmanov E., Baraznenok V., Henderson I., Vrang L., Danielson U.H. (2013). Resolution of the interaction mechanisms and characteristics of non-nucleoside inhibitors of hepatitis C virus polymerase. Antivir. Res..

[81] Mosley R.T., Edwards T.E., Murakami E., Lam A.M., Grice R.L., Du J., Sofia M.J., Furman P.A., Otto M.J. (2012). Structure of Hepatitis C Virus Polymerase in Complex with Primer-Template RNA. J. Virol..

[82] Tomei L., Altamura S., Bartholomew L., Bisbocci M., Bailey C., Bosserman M., Cellucci A., Forte E., Incitti I., Orsatti L. (2004). Characterization of the Inhibition of Hepatitis C Virus RNA Replication by Nonnucleosides. J. Virol..

[83] Takahashi H., Takahashi C., Moreland N.J., Chang Y.-T., Sawasaki T., Ryo A., Vasudevan S.G., Suzuki Y., Yamamoto N. (2012). Establishment of a robust dengue virus NS3–NS5 binding assay for identification of protein–protein interaction inhibitors. Antivir. Res..

[84] Chen C.J., Kuo M.D., Chien L.J., Hsu S.L., Wang Y.M., Lin J.H. (1997). RNA-protein interactions: Involvement of NS3, NS5, and 3’ noncoding regions of Japanese encephalitis virus genomic RNA. J. Virol..

[85] Kapoor M., Zhang L., Ramachandra M., Kusukawa J., Ebner K.E., Padmanabhan R. (1995). Association between NS3 and NS5 Proteins of Dengue Virus Type 2 in the Putative RNA Replicase Is Linked to Differential Phosphorylation of NS5. J. Biol. Chem..

[86] Johansson M., Brooks A.J., Jans D.A., Vasudevan S.G. (2001). A small region of the dengue virus-encoded RNA-dependent RNA polymerase, NS5, confers interaction with both the nuclear transport receptor importin-β and the viral helicase, NS3. J. Gen. Virol..

[87] Singh J., Petter R.C., Baillie T.A., Whitty A. (2011). The resurgence of covalent drugs. Nat. Rev. Drug Discov..

[88] Gehringer M., Laufer S.A. (2019). Emerging and Re-Emerging Warheads for Targeted Covalent Inhibitors: Applications in Medicinal Chemistry and Chemical Biology. J. Med. Chem..

[89] McDonald A.G., Tipton K.F. (2020). Enzymes: Irreversible Inhibition. eLS.

[90] Coleman C.I., Limone B., Sobieraj D.M., Lee S., Roberts M.S., Kaur R., Alam T. (2012). Dosing Frequency and Medication Adherence in Chronic Dis. JMCP.

[91] Bauer R.A. (2015). Covalent inhibitors in drug discovery: From accidental discoveries to avoided liabilities and designed therapies. Drug Discov. Today.

[92] Sutanto F., Konstantinidou M., Dömling A. (2020). Covalent inhibitors: A rational approach to drug discovery. RSC Med. Chem..

[93] Van Arnum P. Drug Repurposing and Repositioning: Making New Out of Old. https://www.dcatvci.org/11-value-chain-insights/114-drug-repurposing-and-repositioning-making-new-out-of-old#.

[94] Rudrapal M., Khairnar S.J., Jadhav A.G. (2020). Drug Repurposing (DR): An Emerging Approach in Drug Discovery. Drug Repurposing.

[95] Ghofrani H.A., Osterloh I.H., Grimminger F. (2006). Sildenafil: From angina to erectile dysfunction to pulmonary hypertension and beyond. Nat. Rev. Drug Discov..

[96] Singh T.U., Parida S., Lingaraju M.C., Kesavan M., Kumar D., Singh R.K. (2020). Drug repurposing approach to fight COVID-19. Pharm. Rep..

[97] Serafin M.B., Bottega A., Foletto V.S., da Rosa T.F., Hörner A., Hörner R. (2020). Drug repositioning is an alternative for the treatment of coronavirus COVID-19. Int. J. Antimicrob. Agents.

[98] Elfiky A.A. (2020). Anti-HCV, nucleotide inhibitors, repurposing against COVID-19. Life Sci..

[99] Sacramento C.Q., de Melo G.R., de Freitas C.S., Rocha N., Hoelz L.V.B., Miranda M., Fintelman-Rodrigues N., Marttorelli A., Ferreira A.C., Barbosa-Lima G. (2017). The clinically approved antiviral drug sofosbuvir inhibits Zika virus replication. Sci. Rep..

[100] Szabat M., Lorent D., Czapik T., Tomaszewska M., Kierzek E., Kierzek R. (2020). RNA Secondary Structure as a First Step for Rational Design of the Oligonucleotides towards Inhibition of Influenza A Virus Replication. Pathogens.

[101] Kesy J., Patil K.M., Kumar S.R., Shu Z., Yong H.Y., Zimmermann L., Ong A.A.L., Toh D.-F.K., Krishna M.S., Yang L. (2019). A Short Chemically modified dsRNA-Binding PNA (dbPNA) Inhibits Influenza Viral Replication by Targeting Viral RNA Panhandle Structure. Bioconjugate Chem..

[102] Wu Y.-S., Lin W.-H., T-A Hsu J., Hsieh H.-P. (2006). Antiviral Drug Discovery against SARS-CoV. Curr. Med. Chem..

[103] He R., Adonov A., Traykova-Adonova M., Cao J., Cutts T., Grudesky E., Deschambaul Y., Berry J., Drebot M., Li X. (2004). Potent and selective inhibition of SARS coronavirus replication by aurintricarboxylic acid. Biochem. Biophys. Res. Commun..

[104] Yap Y., Zhang X., Andonov A., He R. (2005). Structural analysis of inhibition mechanisms of Aurintricarboxylic Acid on SARS-CoV polymerase and other proteins. Comput. Biol. Chem..

[105] Totura A.L., Bavari S. (2019). Broad-spectrum coronavirus antiviral drug discovery. Expert Opin. Drug Discov..

[106] Falzarano D., de Wit E., Martellaro C., Callison J., Munster V.J., Feldmann H. (2013). Inhibition of novel β coronavirus replication by a combination of interferon-α2b and ribavirin. Sci. Rep..

[107] Hart B.J., Dyall J., Postnikova E., Zhou H., Kindrachuk J., Johnson R.F., Olinger G.G., Frieman M.B., Holbrook M.R., Jahrling P.B. (2014). Interferon-β and mycophenolic acid are potent inhibitors of Middle East respiratory syndrome coronavirus in cell-based assays. J. Gen. Virol..

[108] Falzarano D., de Wit E., Rasmussen A.L., Feldmann F., Okumura A., Scott D.P., Brining D., Bushmaker T., Martellaro C., Baseler L. (2013). Treatment with interferon-α2b and ribavirin improves outcome in MERS-CoV–infected rhesus macaques. Nat. Med..

[109] Ferron F., Subissi L., Silveira De Morais A.T., Le N.T.T., Sevajol M., Gluais L., Decroly E., Vonrhein C., Bricogne G., Canard B. (2018). Structural and molecular basis of mismatch correction and ribavirin excision from coronavirus RNA. Proc. Natl. Acad. Sci. USA.

[110] Siegel D., Hui H.C., Doerffler E., Clarke M.O., Chun K., Zhang L., Neville S., Carra E., Lew W., Ross B. (2017). Discovery and Synthesis of a Phosphoramidate Prodrug of a Pyrrolo[2,1-f][triazin-4-amino] Adenine C-Nucleoside (GS-5734) for the Treatment of Ebola and Emerging Viruses. J. Med. Chem..

[111] Warren T.K., Jordan R., Lo M.K., Ray A.S., Mackman R.L., Soloveva V., Siegel D., Perron M., Bannister R., Hui H.C. (2016). Therapeutic efficacy of the small molecule GS-5734 against Ebola virus in rhesus monkeys. Nature.

[112] Sheahan T.P., Sims A.C., Graham R.L., Menachery V.D., Gralinski L.E., Case J.B., Leist S.R., Pyrc K., Feng J.Y., Trantcheva I. (2017). Broad-spectrum antiviral GS-5734 inhibits both epidemic and zoonotic coronaviruses. Sci. Transl. Med..

[113] De Wit E., Feldmann F., Cronin J., Jordan R., Okumura A., Thomas T., Scott D., Cihlar T., Feldmann H. (2020). Prophylactic and therapeutic remdesivir (GS-5734) treatment in the rhesus macaque model of MERS-CoV infection. Proc. Natl. Acad. Sci. USA.

[114] Gordon C.J., Tchesnokov E.P., Woolner E., Perry J.K., Feng J.Y., Porter D.P., Götte M. (2020). Remdesivir is a direct-acting antiviral that inhibits RNA-dependent RNA polymerase from severe acute respiratory syndrome coronavirus 2 with high potency. J. Biol. Chem..

[115] Agostini M.L., Andres E.L., Sims A.C., Graham R.L., Sheahan T.P., Lu X., Smith E.C., Case J.B., Feng J.Y., Jordan R. (2018). Coronavirus Susceptibility to the Antiviral Remdesivir (GS-5734) Is Mediated by the Viral Polymerase and the Proofreading Exoribonuclease. MBio.

[116] Eastman R.T., Roth J.S., Brimacombe K.R., Simeonov A., Shen M., Patnaik S., Hall M.D. (2020). Remdesivir: A Review of Its Discovery and Development Leading to Emergency Use Authorization for Treatment of COVID-19. ACS Cent Sci..

[117] Li G., Clercq E.D. (2020). Therapeutic options for the 2019 novel coronavirus (2019-nCoV). Nat. Rev. Drug Discov..

[118] Beigel J.H., Tomashek K.M., Dodd L.E., Mehta A.K., Zingman B.S., Kalil A.C., Hohmann E., Chu H.Y., Luetkemeyer A., Kline S. (2020). Remdesivir for the Treatment of Covid-19—Final Report. N. Engl. J. Med..

[119] Grein J., Ohmagari N., Shin D., Diaz G., Asperges E., Castagna A., Feldt T., Green G., Green M.L., Lescure F.-X. (2020). Compassionate Use of Remdesivir for Patients with Severe Covid-19. N. Engl. J. Med..

[120] Shannon A., Selisko B., Le N., Huchting J., Touret F., Piorkowski G., Fattorini V., Ferron F., Decroly E., Meier C. (2020). Favipiravir strikes the SARS-CoV-2 at its Achilles heel, the RNA polymerase. Biorxiv.

[121] Agrawal U., Raju R., Udwadia Z.F. (2020). Favipiravir: A new and emerging antiviral option in COVID-19. Med. J. Armed Forces India.

[122] Götte M., Feld J.J. (2016). Direct-acting antiviral agents for hepatitis C: Structural and mechanistic insights. Nat. Rev. Gastroenterol. Hepatol..

[123] Lim S.P., Noble C.G., Seh C.C., Soh T.S., Sahili A.E., Chan G.K.Y., Lescar J., Arora R., Benson T., Nilar S. (2016). Potent Allosteric Dengue Virus NS5 Polymerase Inhibitors: Mechanism of Action and Resistance Profiling. PLoS Pathog..

[124] Noble C.G., Lim S.P., Arora R., Yokokawa F., Nilar S., Seh C.C., Wright S.K., Benson T.E., Smith P.W., Shi P.-Y. (2016). A Conserved Pocket in the Dengue Virus Polymerase Identified through Fragment-based Screening. J. Biol. Chem..

[125] Gharbi-Ayachi A., Santhanakrishnan S., Wong Y.H., Chan K.W.K., Tan S.T., Bates R.W., Vasudevan S.G., El Sahili A., Lescar J. (2020). Non-Nucleoside Inhibitors of Zika virus RNA-dependent RNA polymerase. J. Virol..

[126] Yi D., Li Q., Pang L., Wang Y., Zhang Y., Duan Z., Liang C., Cen S. (2020). Identification of a Broad-Spectrum Viral Inhibitor Targeting a Novel Allosteric Site in the RNA-Dependent RNA Polymerases of Dengue Virus and Norovirus. Front. Microbiol..

[127] Cannalire R., Ki Chan K.W., Burali M.S., Gwee C.P., Wang S., Astolfi A., Massari S., Sabatini S., Tabarrini O., Mastrangelo E. (2020). Pyridobenzothiazolones Exert Potent Anti-Dengue Activity by Hampering Multiple Functions of NS5 Polymerase. ACS Med. Chem. Lett..

[128] Wilkins T., Malcolm J.K., Raina D., Schade R.R. (2010). Hepatitis C: Diagnosis and Treatment. AFP.

[129] Yee B.E., Nguyen N.H., Zhang B., Lin D., Vutien P., Wong C.R., Lutchman G.A., Nguyen M.H. (2015). Sustained virological response and its treatment predictors in hepatitis C virus genotype 4 compared to genotypes 1, 2, and 3: A meta-analysis. BMJ Open Gastroenterol..

[130] Pan Q., Peppelenbosch M.P., Janssen H.L., de Knegt R.J. (2012). Telaprevir/boceprevir era: From bench to bed and back. World J. Gastroenterol..

[131] Pawlotsky J.-M., Feld J.J., Zeuzem S., Hoofnagle J.H. (2015). From non-A, non-B hepatitis to hepatitis C virus cure. J. Hepatol..

[132] McConachie S.M., Wilhelm S.M., Kale-Pradhan P.B. (2016). New direct-acting antivirals in hepatitis C therapy: A review of sofosbuvir, ledipasvir, daclatasvir, simeprevir, paritaprevir, ombitasvir and dasabuvir. Expert Rev. Clin. Pharmacol..

[133] Maasoumy B., Port K., Markova A.A., Serrano B.C., Rogalska-Taranta M., Sollik L., Mix C., Kirschner J., Manns M.P., Wedemeyer H. (2013). Eligibility and Safety of Triple Therapy for Hepatitis C: Lessons Learned from the First Experience in a Real World Setting. PLoS ONE.

[134] Kim A. (2016). Hepatitis C Virus. Ann. Intern. Med..

[135] Wyles D.L., Luetkemeyer A.F. (2017). Understanding Hepatitis C Virus Drug Resistance: Clinical Implications for Current and Future Regimens. Top Antivir. Med..

[136] Liu Y., Lim B.H., Jiang W.W., Flentge C.A., Hutchinson D.K., Madigan D.L., Randolph J.T., Wagner R., Maring C.J., Kati W.M. (2012). Identification of aryl dihydrouracil derivatives as palm initiation site inhibitors of HCV NS5B polymerase. Bioorganic Med. Chem. Lett..

[137] Kati W., Koev G., Irvin M., Beyer J., Liu Y., Krishnan P., Reisch T., Mondal R., Wagner R., Molla A. (2015). In Vitro Activity and Resistance Profile of Dasabuvir, a Nonnucleoside Hepatitis C Virus Polymerase Inhibitor. Antimicrob. Agents Chemother..

[138] Gentles R.G., Ding M., Bender J.A., Bergstrom C.P., Grant-Young K., Hewawasam P., Hudyma T., Martin S., Nickel A., Regueiro-Ren A. (2014). Discovery and Preclinical Characterization of the Cyclopropylindolobenzazepine BMS-791325, A Potent Allosteric Inhibitor of the Hepatitis C Virus NS5B Polymerase. J. Med. Chem..

[139] Lazerwith S.E., Lew W., Zhang J., Morganelli P., Liu Q., Canales E., Clarke M.O., Doerffler E., Byun D., Mertzman M. (2014). Discovery of GS-9669, a Thumb Site II Non-Nucleoside Inhibitor of NS5B for the Treatment of Genotype 1 Chronic Hepatitis C Infect. J. Med. Chem..

[140] Li H., Tatlock J., Linton A., Gonzalez J., Jewell T., Patel L., Ludlum S., Drowns M., Rahavendran S.V., Skor H. (2009). Discovery of (R)-6-Cyclopentyl-6-(2-(2,6-diethylpyridin-4-yl)ethyl)-3-((5,7-dimethyl-[1,2,4]triazolo[1,5-a]pyrimidin-2-yl)methyl)-4-hydroxy-5,6-dihydropyran-2-one (PF-00868554) as a Potent and Orally Available Hepatitis C Virus Polymerase Inhibitor. J. Med. Chem..

[141] Jiao P., Xue W., Shen Y., Jin N., Liu H. (2014). Understanding the drug resistance mechanism of hepatitis C virus NS5B to PF-00868554 due to mutations of the 423 site: A computational study. Mol. BioSyst..

[142] Gentile I., Buonomo A.R., Zappulo E., Borgia G. (2015). Discontinued drugs in 2012–2013: Hepatitis C virus infection. Expert Opin. Investig. Drugs.

[143] Larrey D., Lohse A.W., Trepo C., Bronowicki J.-P., Arastéh K., Bourlière M., Calleja J.L., Stern J.O., Nehmiz G., Abdallah N. (2013). Antiviral Effect, Safety, and Pharmacokinetics of Five-Day Oral Administration of Deleobuvir (BI 207127), an Investigational Hepatitis C Virus RNA Polymerase Inhibitor, in Patients with Chronic Hepatitis C. Antimicrob Agents Chemother..

[144] Kapelusznik L., Heil E.L., Temesgen Z., Talwani R. (2012). Setrobuvir: RNA-directed RNA polymerase inhibitor treatment of hepatitis C virus infection. Drugs Future.

